# The Use of Best Practice in the Treatment of a Complex Diabetic Foot Ulcer: A Case Report

**DOI:** 10.3390/healthcare4010018

**Published:** 2016-03-04

**Authors:** Melodie Blakely

**Affiliations:** The Wound Healing Center of Osceola Regional Medical Center, Kissimmee, FL 34741, USA; melodieblakely@gmail.com; Tel.: +1-206-355-7957

**Keywords:** wound, wound care, diabetes, offloading, unweighting, diabetic ulcer, total contact cast, best practice, felt offloading, multi-disciplinary

## Abstract

*Background and Purpose*: Published guidelines for effective management of diabetic foot ulcers (DFU) include total contact casting (TCC). The purpose of this case study is to describe the application of best practice guidelines for the treatment of a diabetic foot ulcer (DFU) in a complex patient where TCC offloading could not be utilized. *Case Description:* The patient was a 47 year-old female with a five-plus year history of a full-thickness DFU on the left plantar mid-foot. Treatment included sharp and ultrasound debridement, the use of a silver hydrofiber dressing, edema management via compression therapy, negative pressure wound therapy, offloading via customized 1/4 inch adhesive-backed felt applied to the plantar foot in addition to an offloading boot and use of a wheelchair, patient education regarding diabetes management, and the application of a bilayered living skin-equivalent biologic dressing. *Outcomes:* At 15 weeks the wound was closed and the patient was transitioned into diabetic footwear. *Discussion:* The felt offloading was a beneficial alternative to TCC. The patient’s longer than average healing rate may have been complicated by the duration of her wound, her 41 year history of diabetes, and the fact that gold standard offloading (TCC) was not able to be used. Further research is needed regarding the use of felt for offloading, such as application technique for wounds on different areas of the foot, comparison of different types of felt, and the use of felt in conjunction with various offloading devices.

## 1. Introduction

The Centers for Disease Control and Prevention (CDC) reports the incidence of diabetes nationally to be 9.3%, or 29.1 million people. As of 2010, diabetes was the seventh leading cause of death. Those with the disease were 1.5 times more likely to die from diabetes-related complications than those without diagnosed diabetes. Total diabetes-related costs in the United States in 2012 were estimated to be 245 billion dollars, with 176 billion of this in direct medical costs and 69 billion related to disability, work loss, and premature death [1].

Along with cardiovascular and kidney disease, neuropathy is a serious complication of diabetes [1]. Up to 50% of older patients with diabetes show loss of protective sensation (LOPS) in their lower extremities [2]. The pathway to ulceration for diabetic patients is described by Reiber *et al.* [3]. Sensory loss interferes with protective response to noxious stimuli. Motor neuropathy affects the intrinsic muscles of the foot and can lead to deformity and abnormal pressures over bony prominences. These together can lead to repetitive micro trauma and the development of diabetic foot ulcers (DFUs). Risk factors for DFU include previous amputation, past foot ulcer history, peripheral neuropathy, foot deformity, peripheral vascular disease, visual impairment, diabetic nephropathy, poor glycemic control, and cigarette smoking [4]. Peripheral artery disease is prevalent in those with diabetes and has been reported to be a significant contributing factor in the development of DFUs in up to 50% of patients [5]. The lifetime risk of a person with diabetes developing a foot ulcer was reported by Singh *et al*. [6] to be as high as 25%.

Neuropathy and ulceration complicated by delayed healing and/or ischemia are known causative factors leading to amputation [7,8]. Margolis *et al*. [9] found that 4.9% to 5.3% of those with DFUs went on to have a lower extremity amputation. The CDC reports 60% of all non-traumatic lower extremity amputations are associated with diabetes [1]. Post amputation, the 5-year mortality rate is between 39% and 68%, more than many cancers [10,11].

Wound infection in the population with DFU is of particular concern. Approximately 56% of DFUs become clinically infected, greatly increasing risk of hospitalization and amputation [12,13].

Published guidelines and consensus statements on DFU treatment are consistent. International Best Practice Guidelines: Wound Management in Diabetic Foot Ulcers [14] states that the most effective care of DFUs involves a holistic approach and must include optimal diabetes control, effective local wound care, infection control, pressure relieving strategies, and optimization of blood flow. Brem *et al*. [15] recommend optimal glucose control; surgical debridement of all hyperkeratotic, infected, or nonviable tissue; systemic antibiotics for deep infection, drainage, and cellulitis; offloading; moist wound environment; and treatment with growth factors and/or cellular therapy if the wound is not healing in two weeks. Wu *et al*. [16] describe the importance of adequate perfusion, debridement, infection control, and pressure mitigation, and suggest that patient education, optimized glycemic control, smoking cessation, and foot care are also helpful interventions. Jeffcoate *et al*. [17] state principals of DFU management to be treatment of infection, optimization of blood flow, minimization of forces applied to the ulcer, and improvement of the wound condition through wound bed preparation, topical applications, and removal of callus. International Working Group on the Diabetic Foot (IWGDF) guidelines [18] recommend pressure relief and protection of the ulcer, restoration of skin perfusion, management of infection, metabolic control, treatment of co-morbidities such as edema and malnutrition, local wound care including frequent inspection, sharp debridement, and dressings that manage exudates and maintain a moist wound environment, and education for patients and caregivers about appropriate self-care. The IWDGF‘s specific offloading recommendations are that pressure relief should preferably be done with a non-removable, knee-high device such as a total contact cast (TCC), but if this is contraindicated, other types of offloading can be used, such as removable knee-high devices, offloading footwear, and felted foam with appropriate footwear.

The purpose of this case study is to describe the application of best practice guidelines for the treatment of DFUs in a complex patient where TCC offloading could not be utilized.

## 2. Case Description

The patient was a 47 year-old female who was 5 ft. tall and 175 lbs. with a body mass index of 34. She had a five-plus year history of a full-thickness DFU on the left plantar mid-foot. Her DFU had been managed by her podiatrist throughout its course and the patient reported that the wound had been open continuously during this time. Previous interventions included reconstructive surgery for Charcot foot deformity of the left foot six years previously, regular sharp debridement, use of a platelet-derived growth factor (Becaplermin Gel, 0.01%) topically, offloading via total contact casting initially and the use of an offloading walker boot and wheelchair more recently. Reportedly, TCC was discontinued because the patient felt unsteady and declined further use. Other past medical history was significant for Type 1 diabetes mellitus (DM) for 41 years, left eye blindness due to diabetic retinopathy, hypothyroidism and hypertension (HTN). She had a history of cataract surgery on her right eye two years previously. Medications included Synthroid for hypothyroidism, Lisinopril for HTN, and Humulin-N and insulin for DM. The patient was a home-maker and resided with her husband and their three children in a one-level home. She denied use of tobacco or alcohol. She was frustrated by her lack of progress toward wound healing and self-referred to the outpatient wound clinic in order to obtain a second opinion.

## 3. Examination

The patient presented to the out-patient wound center with her husband. She was using a wheelchair and wearing a diabetic walker boot. Her vital signs were assessed in sitting, revealing a blood pressure of 126/60 mmHg, a pulse of 88 beats per minute and a respiratory rate of 18 cycles per minute. No communication barriers were noted; she was oriented to person, place and time. No learning barriers were identified. She reported learning best by listening and demonstration. Her right shoe and left boot were removed. She was noted to have bilateral Charcot foot deformity. The gauze wound dressings were removed and were saturated with a moderate amount of serous drainage. No odor was noted. Her wound measured 13 × 9 mm with 7 mm depth and 7 mm undermining circumferentially. Significant periwound callus and rolled wound edges were present. The wound base was 100% slough and no periwound erythema or induration was noted. No clinical signs of infection were present.

## 4. Tests and Measures

Protective sensation was absent via 10 g monofilament testing over the plantar 1st, 3rd, and 5th metatarsals and great toe of each foot. Sensation testing using a 10 g monofilament has been associated with loss of large-fiber nerve function and has been shown to be predictive of subsequent DFU development [19,20].

Bilateral dorsalis pedis and posterior tibial pulses were easily palpable and were biphasic via hand held Doppler (HHD)™ (Huntleigh, Cardiff, UK). Biphasic sounds signify somewhat decreased vessel elasticity but are considered within normal limits for the distal extremities of adults [21,22]. The classification of blood flow based on Doppler audio output, used in conjunction with other diagnostic techniques such as ankle brachial pulse index (ABPI), is a reliable method for helping the clinician assess for the presence of PAD, determine patient risk status, and monitor vascular status over time [21,22].

Systolic HHD pressure readings were right arm 120, left arm 122, right ankle 116, and left ankle 112. Each of the ankle pressures was divided by the highest arm pressure, respectively, for a right ABPI of 0.92 and a left of 0.95. Widely accepted interpretation of the ABPI is that a value of 1 to 0.8 signifies no significant arterial disease, below 0.8 and greater than 0.5 signifies moderate disease, 0.5 signifies severe disease and 0.3 or below signifies critical ischemia [23].

Edema measurement was completed based on the scale described by O’Sullivan *et al*. [24], where 1+ is a barely detectable impression when a finger is pressed into skin. Bilateral lower extremity edema was noted to be 2+, a slight indentation that takes 15 s to rebound.

Her initial Bates-Jensen Wound Assessment Tool (BWAT) score was 40. The scores range from 13 to 65, 13 signifying wound regeneration and 60 representing wound degeneration. The tool is particularly helpful for showing wound status change over time. The tool was validated for use with pressure ulcers (intrarater reliability averaged 0.89) but has been modified for use with all wound types [25,26].

## 5. Evaluation

The physical therapy diagnosis was full-thickness diabetic foot ulcer—Wagner grade 1 [27]. The preferred practice pattern according to the Guide to Practice for the Physical Therapist [28] was Integumentary Pattern 7D: Impaired integumentary integrity associated with full-thickness skin involvement and scar formation. Short term goals were to detect and manage infection, effectively offload the wound area, remove non-viable and hyperkeratotic tissue, maintain a moist wound environment, and promote 50% reduction in wound size within 8 weeks.

Long term goals were to achieve complete epithelialization within 16 weeks, transition the patient to custom diabetic footwear, increase patient/family understanding of the disease process, and encourage follow up with the primary care provider (PCP), endocrinologist, and podiatrist. The frequency and duration of treatment was set at one time per week for 16 weeks.

The patient’s prognosis for wound healing was good due to adequate blood flow and good family support.

## 6. Intervention

**Figure 1:**
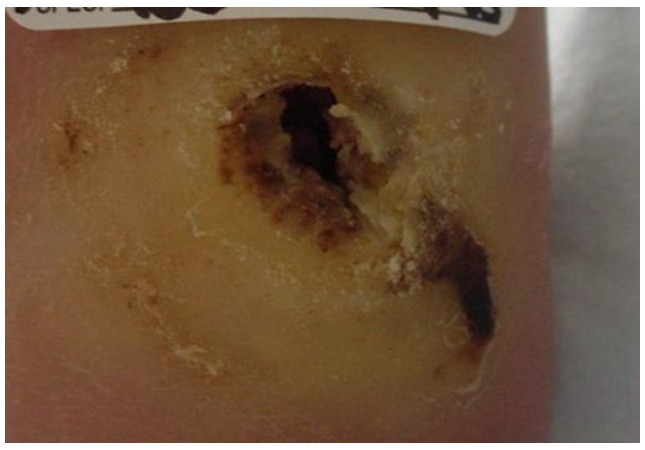
Wound appearance at initial visit.

At the initial visit (Figure 1) sharp debridement with curette, scalpel, and forceps was performed to remove slough and periwound callus. The wound was dressed with AQUACEL^®^ Ag (ConvaTec Inc, Greensboro, NC, USA), a silver hydrofiber, gauze 4 × 4s and an ABD pad, and held in place with 4 inch gauze roll. This was to be changed every other day. Tubigrip^®^ (Molnlycke Health Care, Norcross, GA, USA), an elastic tubular bandage from the base of the toes to just below the knee was used for edema management. The diabetic walker boot and wheelchair were continued for offloading. The patient and her husband were given verbal and written instruction regarding local wound care, the importance of using her offloading devices diligently, and how to recognize and respond to signs and symptoms of infection. They were also given verbal instruction and a handout from the Diabetes Care and Education Group regarding diabetes management [29]. Home health nursing services were arranged for mid-week dressing changes.

**Figure 2:**
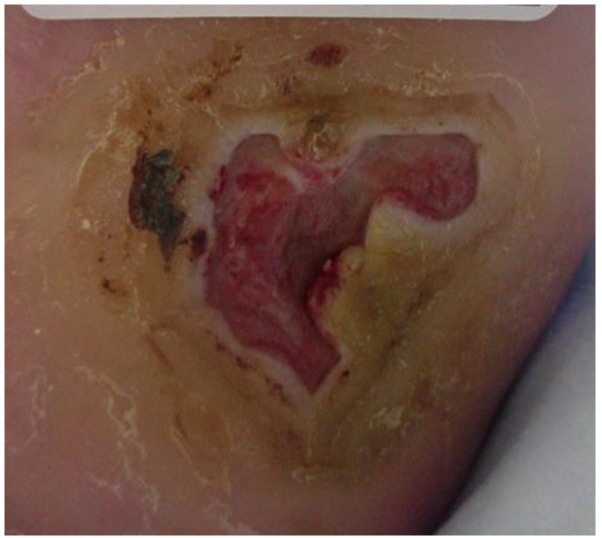
Wound appearance at day 5.

Five days later the wound bed was 60% covered with slough with hyperkeratotic, rolled wound edges (Figure 2). The patient was brought in for ultrasound (US) debridement with the Sonic One^®^ (Misonix, Inc., Farmingdale, NY, USA) US debrider. US at 22.5 kHz was delivered at intensity level 5 (on a scale of 0 to 5), continuous pulse setting for 4.5 min. The wound bed was left free of non-viable tissue. The silver hydrofiber was continued. The patient’s offloading boot was further customized with ¼ inch felt padding, cut to offload the area. Because the wound had been present for greater than 6 months, she was sent for a magnetic resonance imaging (MRI) scan and an x-ray. These were inconclusive but suggestive of possible osteomyelitis. A tagged white blood cell (WBC) scan was ordered. Weekly sharp debridements, every other day silver hydrofiber dressings, and offloading with the walker boot and wheelchair were continued.

**Figure 3:**
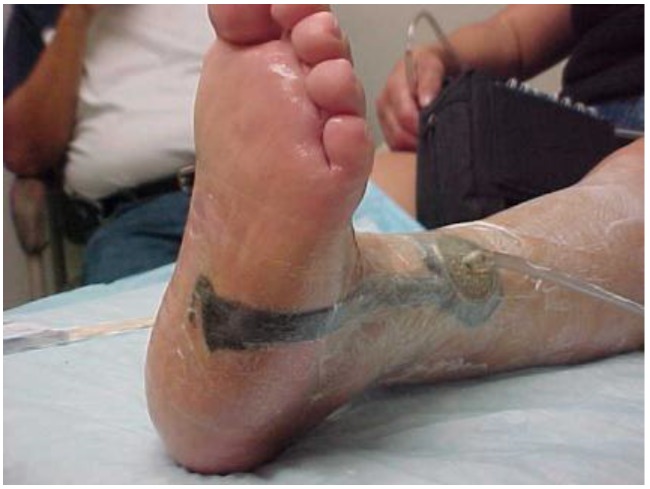
Application of NPWT at week 4.

By week four, results of the WBC scan were obtained and were negative for osteomyelitis. The wound bed was clean and granular but still with significant depth. To better address wound depth, negative pressure wound therapy (NPWT) was initiated using the Vacuum-Assisted Closure (V.A.C.)^®^ (KCI San Antonio, TX, USA) device, modified by bridging the tube attachment pad to the distal leg with foam to avoid tubing pressure on the plantar surface (Figure 3). NPWT settings were 125 mmHg, continuous. This was changed three times a week. Weekly debridements and offloading were continued.

**Figure 4:**
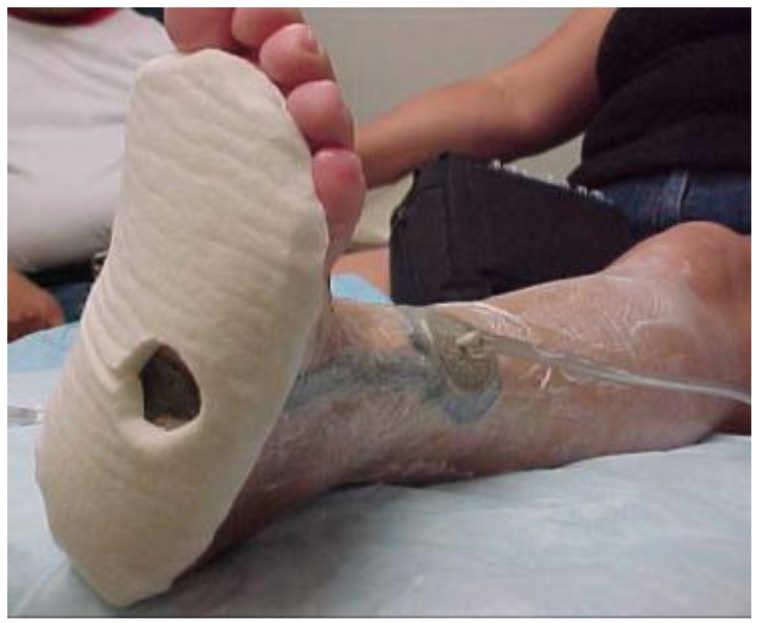
Application of 1/4 inch felt at week seven.

**Figure 5:**
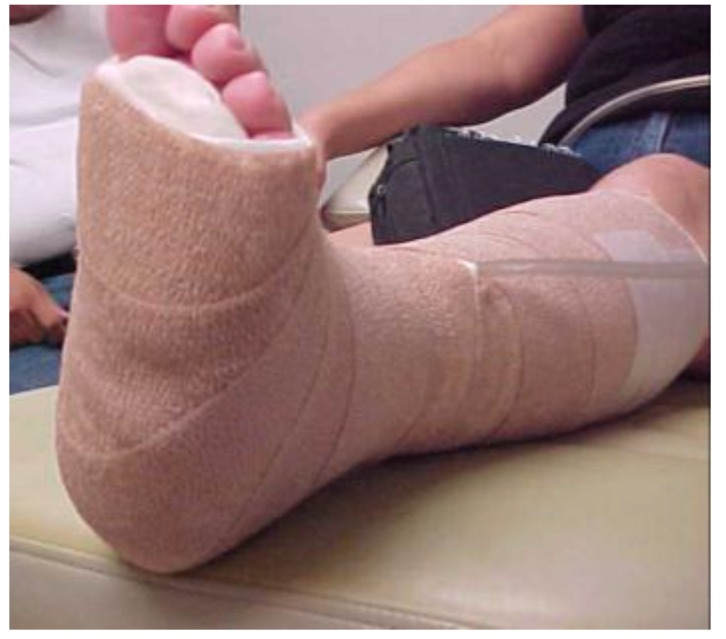
Application of multi-layer wrap at week seven.

**Figure 6:**
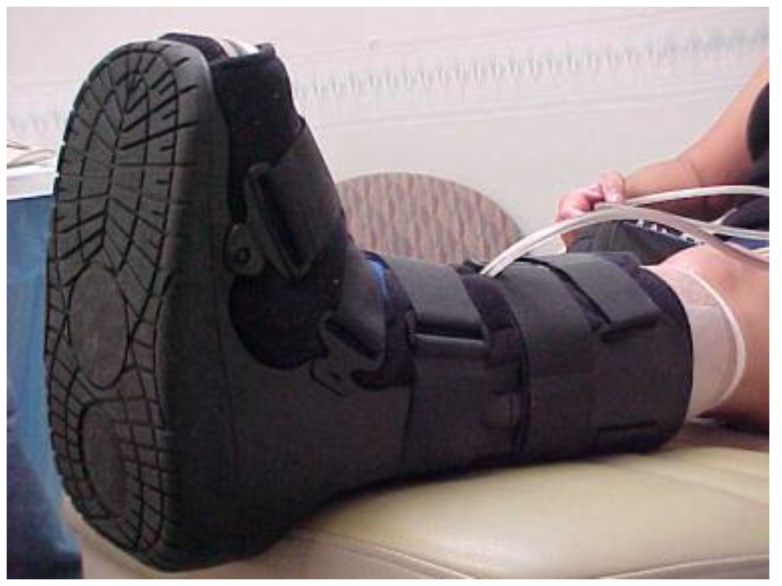
Felt, wrap and boot in place at week seven.

At week seven, the wound bed was clean, with less depth but with persistent rolled wound edges, despite debridement at each visit. It was decided to focus on further possible barriers to healing. The continued rolled wound edges suggested that offloading was not yet optimal. The use of ¼ inch Sammons Preston Orthopedic Felt^®^ (Patterson Medical, Warrenville, IL, USA), an adhesive-backed felt, customized to offload the wound area and applied to the plantar surface of the foot, was initiated to further isolate pressure relief. AllKare Protective Barrier Wipe^®^ (ConvaTec Inc, Greensboro, NC, USA), a skin prep product, was first applied liberally to the entire plantar surface. The negative pressure device was applied as noted earlier. The adhesive-backed felt pad was cut to fit the patient’s plantar foot from the base of the toes to the heel. A relief hole was cut to correspond with the wound area. The felt was then applied directly to the plantar surface with the relief hole exposing the wound area. A DYNA-FLEX^®^ (Systagenix, North Yorkshire, United Kingdom) three-layer compression wrap was then applied to the leg from the base of the toes to just below the knee. It was wrapped so that the negative pressure tubing was outside of the wrap to avoid additional pressure areas. The boot was then applied (Figure 4, Figure 5 and Figure 6). All of this was changed twice a week at the wound clinic.

**Figure 7:**
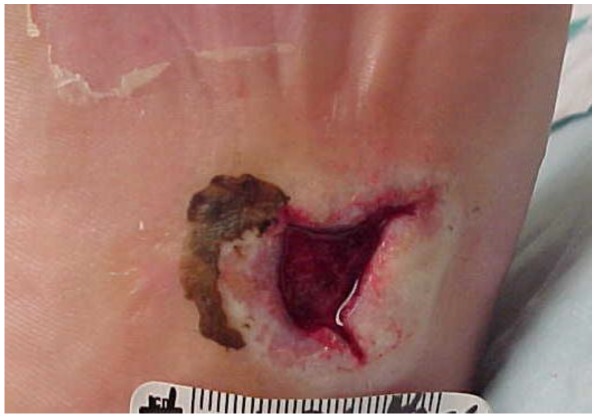
Appearance of wound at week eight, prior to application of bi-layered living skin equivalent.

At week eight (Figure 7), NPWT was discontinued, as the wound depth had filled in with granulation tissue. The wound bed was now well prepared, and Apligraf^®^ (Organogenesis, Canton, MA, USA), a bi-layered living skin equivalent, was applied and held in place with adhesive strips. This was covered with a non-stick contact layer of Mepitel^®^ (Molnlycke Health Care, Norcross, GA, USA). Felt padding was again applied to the plantar surface. Silver hydrofiber and dry gauze were applied to the wound over the cut out portion of the felt. The three-layer wrap and offloading boot were reapplied. The patient returned to the clinic for weekly dressing changes.

**Figure 8:**
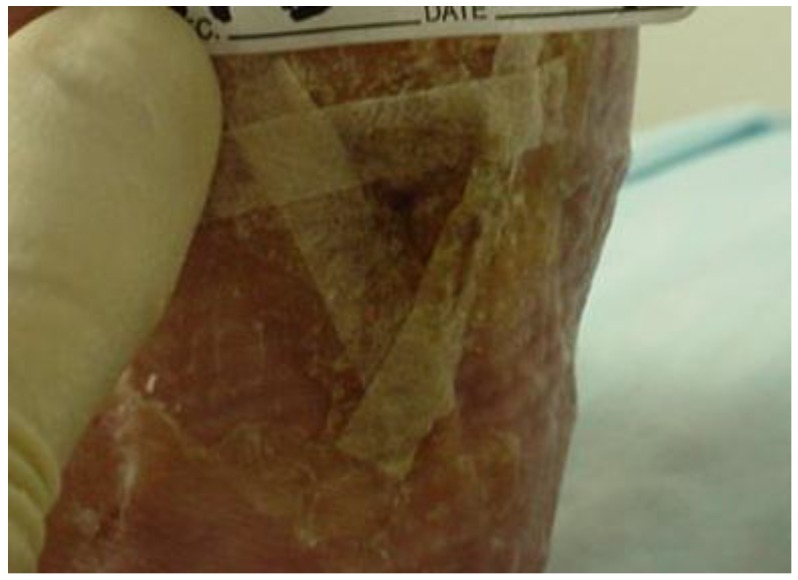
Appearance of wound at week 12, after re-application of bi-layered living skin equivalent.

At week 12, the bi-layered living skin equivalent was re-applied (Figure 8). Dressings, offloading and wraps were continued as before. It had been arranged for a local orthotic company to come to the clinic during the patient visit to mold her feet for new custom molded diabetic shoes in anticipation of approaching wound closure.

**Figure 9:**
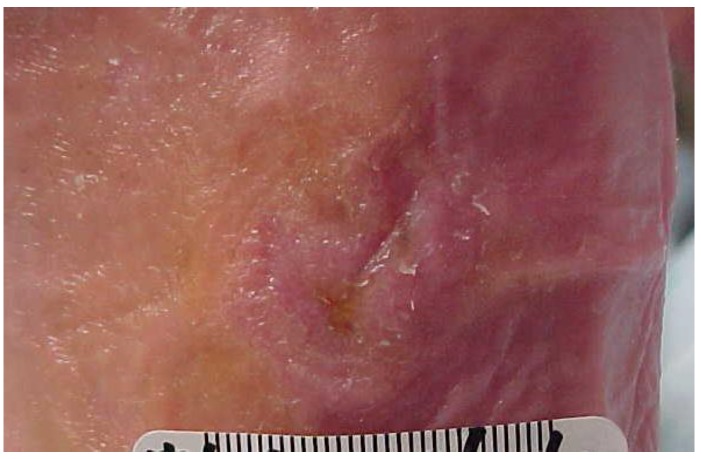
Appearance of wound at week 15.

At week 15, wound closure was achieved (Figure 9) and the patient was transitioned into her new diabetic footwear. She was instructed to gradually increase time in her shoes and to check her feet frequently for areas of redness or skin trauma.

The patient returned for a follow-up visit the following week to ensure a smooth transition to the custom footwear. The wound remained closed. Patient education regarding the importance of using her offloading devices diligently and how to recognize and respond to new wounds or signs and symptoms of infection were reviewed. The American Diabetes Association handout was reviewed. The patient and her husband verbalized understanding. The patient agreed to follow up with her PCP and endocrinologist. She was given names of podiatrists with whom she could follow up and she agreed to seek regular care from the podiatrist of her choice.

## 7. Outcomes

**Figure 10:**
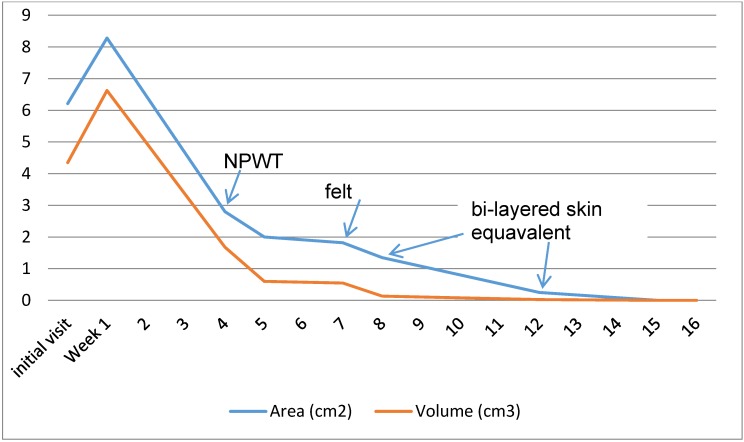
Area and wound measurements over time with NPWT, ¼ inch felt offloading, and bi-layered skin equivalent interventions indicated.

Figure 10 shows the wound measurements across the weeks of treatment. At the initial visit, debridement made the wound significantly larger but created a clean red base and beveled wound edges. Five days later, ultrasound debridement further enlarged the wound. By week four, the wound was smaller, but still exceeded the initial visit measurements, and NPWT was begun. At week five the wound depth had decreased by 50%. At week eight, a week after beginning felt offloading, the wound continued to close, and the bi-layered skin equivalent was applied. At week 12, the wound measured 0.5 × 0.5 mm with 0.1 cm depth; at week 15 the wound was 100% epithelialized and the patient was able to transition to custom molded diabetic footwear. At this point, the patient’s BWAT score had decreased from an initial score of 40 to 15, signifying an epithelialized full-thickness wound. She was transitioned into custom molded diabetic shoes. The wound remained closed at her week 16 follow-up visit. All short and long term goals were met.

## 8. Discussion

Risk factors for DFU include previous amputation, past foot ulcer history, peripheral neuropathy, foot deformity, peripheral vascular disease, visual impairment, diabetic nephropathy, poor glycemic control, and cigarette smoking. Of these, this patient presented with peripheral neuropathy, foot deformity, diabetic neuropathy, and visual impairment. Glycemic control had been an issue at times in her 40-plus years of having diabetes, but was now controlled and under the close supervision of her endocrinologist. Her foot deformity and visual impairment were also quite significant, with complete arch collapse of both feet and complete blindness in one eye. The patient’s medical status was complex and she was at high risk for additional DFUs and amputation. The presence of osteomyelitis greatly increases the risk of lower extremity amputation [12]. Wu *et al*. [16] suggest that education may be an important factor in risk mitigation. Education of the patient and her spouse was an important component of the holistic approach used in this case report, as it provided a better opportunity for the patient to manage those risk factors within her control.

It is generally agreed that the principles of DFU management include diabetes control, effective local wound care, infection control, pressure relieving strategies, and optimization of blood flow. In this case, all of these principles were addressed. Diabetes control was closely monitored by the patient’s endocrinologist, and blood flow was assessed and determined to be adequate.

Effective local wound care in this case followed the principles set forth in the widely accepted Tissue management, Inflammation and infection control, Moisture balance, and Epithelial (edge) advancement (TIME) model, detailed in the European Wound Management Association (EWMA) position document *Wound bed preparation in practice* [30].

Tissue management was achieved through the removal of non-viable tissue, bacteria, and cells that impede the healing process, thus providing an environment that stimulates new tissue growth. Edge advancement was achieved through sharp debridement of hyperkeratotic peri-wound tissue and callus, good local wound care, and protection of the wound from trauma. Studies have shown that regular sharp debridement of the diabetic foot ulcer aids in healing [31,32]. Though the patient reported debridement of her wound by her podiatrist prior to being treated in the wound center, the amount of undermining and peri-wound callus would suggest that wound edge management was not optimal. In the outpatient wound center, the patient underwent weekly debridements to remove any non-viable tissue, cellular debris and hyperkeratotic tissue. At each visit, periwound callus was removed and the wound edges were made beveled, thus promoting epithelial migration.

The patient had no clinical appearance of infection; however, local bioburden was managed with the use of silver hydrofiber dressings. Due to the high incidence of infection in DFUs [33] and the five-plus year duration of the wound, it was felt that it was important to rule out osteomyelitis. This was accomplished through MRI and tagged WBC scanning, along with careful attention to clinical presentation.

According to Teik *et al.* [34], the effect of autonomic neuropathy on sympathetic vascular tone makes those with diabetes more prone to edema. This inhibits nutrient exchange at the capillary level, resulting in impaired wound healing. Edema management is, therefore, an important consideration in the treatment of DFU. Bowering *et al*. [35] found compression therapy to be an effective and safe treatment for the reduction of edema in patients with DFUs. Compression therapy must be done in the context of the patient’s arterial status; in this case the patient’s arterial status allowed for use of compression therapy. This was achieved, initially, through the use of an elastic tubular bandage, applying approximately 8 mmHg of compression. When this was tolerated well, a multi-layer wrap was utilized, applying a more clinically effective level of compression, approximately 30 mmHg [36].

Offloading was achieved through the application of ¼ inch adhesive-backed felt to the plantar surface of the foot and through the use of a diabetic walker boot and a wheelchair. TCC is regarded as the gold standard for offloading DFUs [37]; however, according to Wu *et al*. [38], drawbacks to TCC can include skin irritation, gait instability, and the inability to visualize or treat the wound on a frequent basis if needed. TCC is generally contraindicated in cases involving concomitant soft tissue infection, osteomyelitis, and/or ischemia. In a comparison of different types of offloading devices, it was found that TCC was the most effective offloading device in decreasing peak pressures with ambulation, but that removable cast walkers (RCW) reduced peak pressures only slightly less than TCC [39]. Despite this, it is reported that RCWs have a significantly lower healing rate than TCC [40]. This may be due to compliance with wearing the offloading device when it is able to be removed. Armstrong *et al.* in 2003 [41] found that patients with removable offloading devices only used them 30% of the time. Not surprisingly, a randomized controlled trial by Armstrong *et al*. in 2005 found that non-removable devices were more effective than removable devices in the healing of DFU [42]. The use of felt is not well researched. One study compared the offloading ability of TCC, half shoes, RCWs, rigid postoperative shoes, and felted foam accommodative dressings. The ability of the felt to reduce plantar pressures was less than TCC, RCW, and the half shoe, but was better than the surgical shoe [43]. Further research is needed regarding the use of felt for offloading, such as application technique for wounds on different areas of the foot, comparison of different types of felt, and the use of felt in conjunction with various offloading devices. In this patient’s case, TCC was not utilized for several reasons. The patient verbalized that she felt unsteady when it had been applied in the past and that she did not want to be treated with TCC. In addition, due to the long duration of the wound, the presence or absence of osteomyelitis was not certain. The choice was made to continue the use of the RCW, as the patient was already comfortable with it and felt steady when ambulating. This also allowed more frequent visualization and treatment of the wound. Pressure reduction was further isolated to the area by applying felt directly to the plantar surface of the foot. In addition to improving pressure relief to the wound, it added a non-removable component to the offloading. The multi-layer compression wrap utilized for edema management had the added effect of holding the felt padding in place and further enhancing the non-removable nature of the dressing. A custom molded offloading boot was considered, but the patient’s insurance co-pay was cost prohibitive.

Moisture balance was achieved through the use of appropriate dressings based on wound assessment at each visit.

At week four of treatment the wound bed was clean and free of non-viable tissue, but still had significant depth. Eginton *et al*. [44], in a randomized trial, found that large DFUs treated with NPWT decreased wound volume and depth significantly better than moist gauze dressings. NPWT was initiated in order to stimulate the formulation of granulation tissue and decrease wound volume.

Once granulation tissue had reached the surface of the skin, a bi-layered living skin equivalent was applied. In two large multicenter clinical trials, bi-layered cultured skin equivalent was shown to heal non-infected, non-ischemic chronic plantar diabetic foot ulcers faster and in more patients than conventional therapy [45,46]. Bi-layered living skin equivalent is an effective but expensive therapy. Sheehan *et al*. [47] suggest that advanced therapies such as this should be used when the patient is not on a normal healing trajectory and risk of costly complications outweighs cost of treatment. Advanced therapies were used in this case due to the significant complexity and chronicity of the patient’s wound.

This patient fell somewhat outside the average healing time found in the literature for DFUs. Her wound closed in between 98 and 105 days. Zimmy *et al*. [48] found that in non-ischemic patients with DFUs and appropriate care, the average healing time was 77.7 (95% CI 62–93) days. Her progression does fall in line with findings that those with reduction in ulcer area less than 53% at four weeks had a healing rate of only 9% (*p* < 0.01) at 12 weeks [47]. Her healing rate may have been complicated by the duration of her wound, her 41 year history of diabetes, and the fact that gold standard offloading (TCC) was not able to be used. Patient compliance may have also been a factor. The felt offloading was non-removable; however, if she did not use the RCW 100% of the time at home, the felt alone would have provided sub-optimal offloading.

The importance of a multi-disciplinary approach in DFU care is well established [14,15,16,17,18]. This patient benefitted by being treated in a wound center, as treatments which fell outside of the physical therapy scope of practice, such as the ordering of labs and tests, and those only reimbursed when done by a physician, such as application of a bi-layered living skin equivalent, were coordinated within the wound center team and provided by the appropriate team member.

## 9. Conclusions

This case report describes the application of best practice guidelines in the treatment of a complex DFU where the use of total contact casting was not practical. The patient had been diabetic for more than 40 years with a DFU of greater than 5 years duration prior to presenting to the outpatient wound center. The wound was structurally complicated by Charcot deformity with complete arch collapse. Significant edema was present and protective sensation was absent. Ultrasound debridement, silver hydrofiber, NPWT, a bi-layered skin equivalent, a three-layer wrap system, and offloading techniques which included the use of ¼ inch adhesive felt were utilized to customize treatment. Through attention to diabetes control, optimization of blood flow, infection control, effective local wound care, and pressure relieving strategies, her wound was closed in 15 weeks.
